# Antifungal Mechanism Effect of Artemisinin on *Fusarium solani*

**DOI:** 10.3390/ph18111696

**Published:** 2025-11-08

**Authors:** César Espinoza, Francisco Roberto Quiroz-Figueroa, Oswaldo Guzmán-López, Eliel Ruiz-May, Verónica Gallegos-García, Alejandro Salinas-Castro, Giovanny García-Serrano, Thuluz Meza-Menchaca

**Affiliations:** 1 Centro de Investigación en Micología Aplicada, Universidad Veracruzana, Médicos 5, Unidad del Bosque, Xalapa-Enríquez 91010, Mexico; cespinoza@uv.mx (C.E.); asalinas@uv.mx (A.S.-C.); 2 Instituto Politécnico Nacional, Centro Interdisciplinario de Investigación para el Desarrollo Integral Regional (CIIDIR)—Unidad Sinaloa, Guasave 81101, Mexico; fquirozf@hotmail.com; 3 Facultad de Ciencias Químicas, Universidad Veracruzana, Coatzacoalcos 96523, Mexico; osguzman@uv.mx; 4 Laboratorio de Farmacología, Escuela Militar de Graduados de Sanidad, Universidad del Ejército y Fuerza Aérea, Ciudad de México 11200, Mexico; pelecas40@gmail.com; 5 Escuela de Enfermería y Nutrición, Universidad Autónoma de San Luis Potosí, San Luis Potosí 78290, Mexico; veronica.gallegos@uaslp.mx; 6 Laboratorio de Investigación Médico-Biológica, Facultad de Medicina, Universidad Veracruzana, Médicos y Odontólogos, Virginia Cordero de Murillo Vidal, Xalapa-Enríquez 91017, Mexico; zs23021574@estudiantes.uv.mx

**Keywords:** *Fusarium solani*, artemisinin, reactive oxygen species, antiproliferative, confocal microscopy

## Abstract

**Background:** *Fusarium solani* (*Fs*), a drug-resistant phytopathogenic fungus, is a major cause of severe infections in both plants and humans. *Artemisia annua* and its derivatives exhibit antimicrobial, antiviral and anticholesterolemic activities, yet their clinical use has been dominated by potent antimalarial and anticancer effects. Artemisinin (ART), a sesquiterpene lactone isolated from *A. annua*, is well recognized for its antimalarial efficacy but remains underexplored as an antifungal agent. **Methods:** Conidia of *Fs* were treated with increasing concentrations of ART (75–500 μM) for 0 and 24 h. Fungal viability was assessed using viability assays. Membrane permeability was examined using confocal laser scanning microscopy with propidium iodide (PI) staining. Protein carbonylation assays were performed to quantify oxidative damage induced by ART. **Results:** A 24 h, ART exposure reduced *Fs* viability in a dose-dependent manner, with an IC_50_ of 147.5 μM. At 500 μM, ART achieved fungicidal activity with 99% growth inhibition. Confocal microscopy confirmed extensive membrane disruption in ART-treated conidia, while carbonylation assays demonstrated marked protein oxidation, supporting a mechanism involving free radical generation from the peroxide bridge of ART. ART exhibits potent antifungal activity against *Fs*, mediated by oxidative stress, membrane disruption and protein carbonylation. **Conclusions:** These findings highlight ART as a promising candidate for antifungal drug development against resistant *Fusarium* species.

## 1. Introduction

Fusariosis is a well-documented infection in plants by *Fusarium* species, a significant soil-inhabiting phytopathogen causing huge losses in agriculture. Besides affecting crop fields, *Fusarium* spp. are capable of inducing infections in animals and humans, mostly in immunocompromised patients [1]. Ranked among non-dermatophytic pathogenic moulds in the company of *Aspergillus*, *Scopulariopsis*, *Alternaria*, and *Acremonium* spp. *Fusarium* has been associated with varied clinical conditions like onychomycosis [2,3], keratitis [4], and endophthalmitis [5]. Out of many *Fusarium* species, *Fusarium solani* (*Fs*) has the highest isolation frequency in clinical cases and hence is of special biomedical relevance in the present study [6].

*Fusariosis* is identified as one of the two most prevalent mould infections in immunocompromised patients, after *Aspergillus* spp. infections. *Fs* is also one of the most widely dispersed filamentous fungi in terrestrial and aquatic ecosystems. Its ability to rapidly adapt through evolution, possibly by virtue of higher mutation rates participating in DNA repair mismatches [7], enhances its robustness and pathogenic potential. Crucially, recent evidence demonstrates the possibility of *Fs* in allowing cross-kingdom transmission, with even recorded cases of infections being transferred from humans to maize [8]. Others have identified sophisticated fungal–bacterial interactions within infected tomato fruits [9]. Collectively, these reports reveal the ecological versatilities and pathogenic potencies of *Fs* and have important consequences in agriculture and in human health.

Antimicrobial agents are critical in the treatment of infectious diseases caused by pathogenic microorganisms through a range of mechanisms of action. With the fortuitous serendipitous discovery of penicillin in the 1920s, the discovery of antibiotics has revolutionized the science of medicine over the past century. Following up on traditional methods of empiricism in traditional forms of medicine, investigation of *Artemisia annua* L. (Asteraceae) has confirmed considerable antiplasmodial effectiveness in malaria. Through systematic experimental analysis, Chinese researchers investigated the traditional herbal remedy qinghaosu and identified that the plant leaves contain exceptionally high levels of a sesquiterpene lactone defined by an unusual endoperoxide bridge (a seven-membered ring incorporating peroxide). Since entering into clinical therapy, ART has saved millions of lives and significantly enhanced health benefits worldwide [10]. The compound ART has remarkable specificity by alkylating proteins and other macromolecules in target cells and tissues. However, the precise molecular mechanisms underlying its effectiveness are still in part unknown [11].

Previously, our group established that singlet oxygen has the ability to permeabilize lipid bilayers enriched in ergosterol to rupture membranes upon photosensitization. This phenomenon has been demonstrated using *Candida tropicalis* and otherwise verified through atomic force microscopy visualizations of bilayers containing sterol. These results justify an efficient antifungal modality specifically targeting membrane-enriched fungi containing ergosterol in a simple and efficient treatment modality [12].

Considering the distinctive mechanism of action exhibited by artemisinin and the susceptibility of membranes enriched with ergosterol, the current investigation intends to explore the diverse therapeutic attributes of ART in relation to the prevalent fungal pathogen *Fs*. Through the clarification of ART’s antifungal efficacy and possible mechanisms of action, this study aspires to integrate perspectives from plant pathology and antimicrobial treatment, thus promoting novel approaches to address infections caused by *Fs*.

## 2. Results

### 2.1. Cytotoxicity Assay

**Figure 1:**
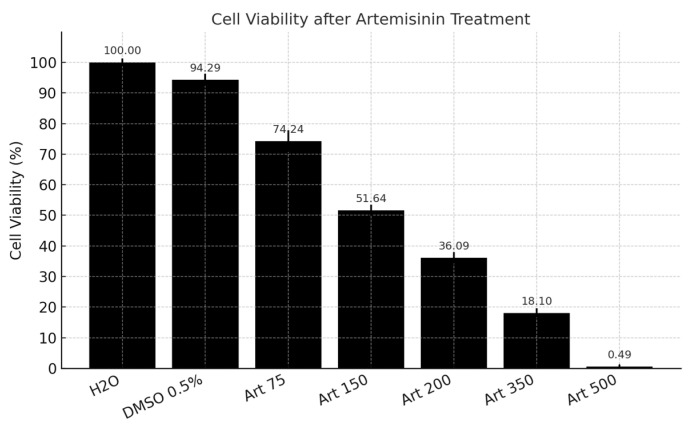
Viability of *Fs* conidia following ART treatment. ART treatment showed a progressive, concentration-dependent reduction in cell viability. Negative controls (H_2_O and 0.5% dimethyl sulfoxide [DMSO]) are shown on the left for reference. The data were analyzed using a one-way analysis of variance (ANOVA), with Tukey’s post hoc test using Mitinab 19 software, and a significance level of 0.05. Different letters indicate statistically significant differences between treatments.

ART treatments against *Fs* conidia revealed stable cell viability in non-exposed groups (95–99%), with minimal reductions across controls and lower ART concentrations. However, following 24 h exposure, a concentration-dependent decrease in cell viability was observed. The calculated IC_50_ for ART was 147.5 μM (Figure 1).

Growth inhibition assays demonstrated that ART exerted a fungicidal effect at 500 μM, achieving 99% inhibition of *Fs*. At lower concentrations (350, 200, and 150 μM), ART displayed fungistatic activity, corresponding to inhibition rates of 82, 64 and 48%, respectively, after 24 h of expo-sure. Following 24 h exposure of *Fs* conidia to ART at concentrations of 500, 300, and 150 μM, cell viability was assessed. A control group was included using sterile distilled water. To further evaluate membrane integrity, the treated samples were subjected to propidium iodide (PI) staining and analyzed via confocal laser scanning microscopy.

### 2.2. In Situ ART-Induced Membrane Permeability

**Figure 2:**
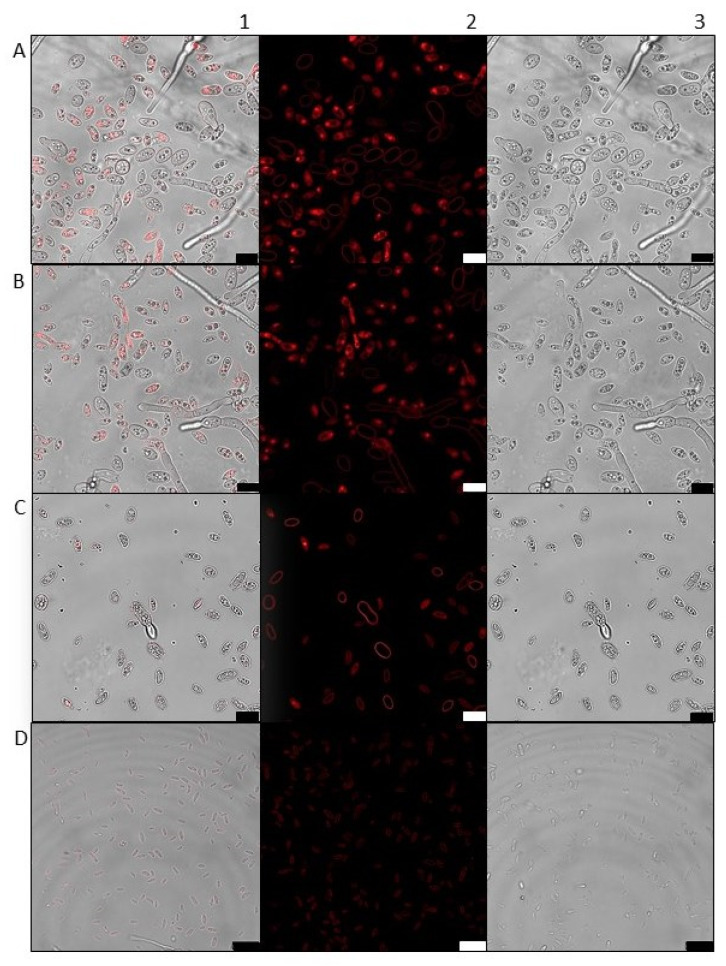
Confocal microscopy of *Fs* conidia stained with propidium iodide (PI) following ART treatment. Panels (**A**–**C**) show conidia exposed to 500, 300, and 150 μM ART, respectively, exhibiting increased red fluorescence consistent with membrane damage and enhanced permeability. Panel (**D**) shows the growth control (sterile H_2_O), with minimal PI uptake and fluorescence restricted to the membrane surface. For each treatment, images are displayed as: (1) overlay, (2) PI fluorescence, and (3) transmitted light. Rows (**A**–**C**): 10 µm scale bar. Row (**D**): 20 µm scale bar.

As stated above, confocal microscopy is connected to reactive oxygen species (ROS) detection, as reactive oxygen species are compartmentalized and mobile, and it is all timed and spatially-dependent upon when they are generated. So, with that said, building on previous cytotoxicity studies of peroxide-based compounds [12], we examined whether ART induced membrane permeability sufficient to permit PI entry a marker of compromised membrane integrity. *Fs* conidia treated with ART exhibited a pronounced increase in red fluorescence, consistent with extensive membrane damage and enhanced permeability (Figure 2). Conidia exposed to 500 μM ART were uniformly PI-positive, showing widespread internal staining and confirming complete membrane disruption. By contrast, control conidia treated with sterile water displayed no intra-cellular PI signal, with fluorescence confined to the membrane periphery.

### 2.3. Oxidative Damage as the Mechanism of ART Fungicidal Activity

**Figure 3:**
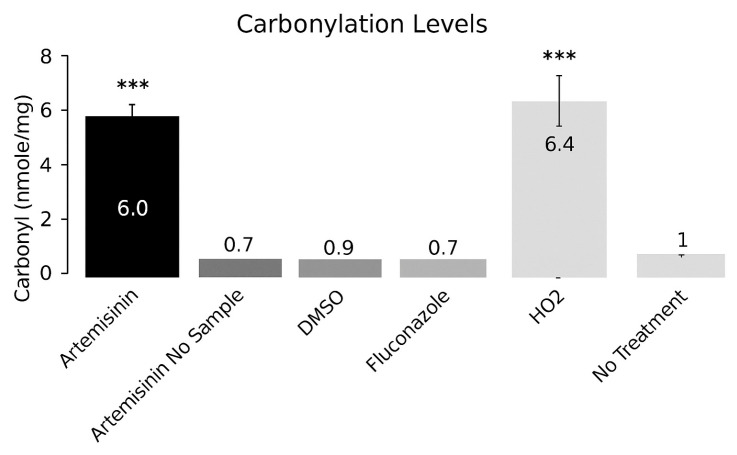
Bar graph showing carbonyl concentrations in *Fs* cells following different treatments. Lane 1, untreated control; Lane 2, <1% dimethyl sulfoxide (DMSO); Lane 3, ergosterol peroxide (40 μM); Lane 4, ergosterol (40 μM); Lane 5, hydrogen peroxide (120 μM); Lane 6, ergosterol peroxide in the absence of cells. All treatments were applied to suspensions containing 10^4^ cells/mL. A statistically significant increase in protein carbonylation was observed in cells treated with ergosterol peroxide (*** *p* ≤ 0.05, one-way non-parametric ANOVA).

The data indicate that ART’s fungicidal properties derive from cleavage of its peroxide bridge, generating ROS that inflict oxidative damage on lipids, proteins, and nucleic acids consistent with the membrane permeabilization observed by confocal microscopy. To assess whether this oxidative damage was driven by peroxide bond homolysis, we quantified post-treatment carbonyl levels using a protein carbonylation assay (Figure 3). One-way ANOVA revealed a significant overall effect of treatment on protein carbonylation (F(5, 48) = 26.81, *p* < 0.0001). Post hoc Tukey’s tests confirmed that ART and H_2_O_2_ treatment groups displayed significantly higher carbonylation compared to untreated controls (*p* < 0.001), whereas ergosterol peroxide, DMSO, and fluconazole did not differ significantly from control. A marked increase in carbonylated proteins was detected in cells treated with ergosterol peroxide, relative to untreated controls or vehicle (DMSO) alone.

Together with the concentration-dependent reduction in conidial viability (IC_50_ = 147.5 μM) and the growth inhibition assays demonstrating fungicidal activity at 500 μM and fungistatic effects at lower concentrations, these results provide a coherent mechanistic model. ART acts via peroxide-mediated free radical generation, culminating in oxidative biomolecular injury and ultimately driving fungal growth suppression.

## 3. Discussion

Among the *Fusarium* species studied, *Fs* demonstrated notable resistance to several antifungal agents, including flucytosine, ketoconazole, miconazole, fluconazole, itraconazole, and posaconazole [13]. These findings, combined with the limitations of conventional antifungal therapies such as toxicity, adverse effects, high cost, low biocompatibility, and multidrug resistance highlight the urgent need for the development of novel therapeutic compounds.

Recent studies have evaluated the antifungal potential of *Artemisia annua* extracts against *Fusarium* spp., primarily in the context of agricultural applications targeting pathogenic consortia in plant roots [14,15,16,17,18]. In one such study, extract concentrations as low as 4 μL/mL were effective. This efficacy has been attributed to the extract’s phytochemical composition, which includes camphor, borneol, deoxyartemisinin, and β-caryophyllene oxide [19,20].

However, ART, the principal antimalarial compound derived from *A. annua*, represents only a small fraction, approximately 0.01%, of the plant’s dry biomass [21], presenting scalability challenges for therapeutic use.

Despite this limitation, our findings demonstrate that ART alone is sufficient to exert a significant antifungal effect. While many studies have focused on crude *A. annua* extracts, our results underscore ART as the primary active agent in suppressing *Fs* growth.

Interestingly, among the *Fusarium* spp. studied, *Fs* has been shown to exhibit greater resistance to flucytosine, ketoconazole, miconazole, fluconazole, itraconazole, and posaconazole [22]. Current pharmacological options are limited by drug toxicity, adverse side effects, high costs, low biocompatibility, and multidrug resistance. These issues underscore the importance of re-searching and expanding the repertoire of proven therapeutic compounds available for common diseases.

Earlier reports have documented antifungal activity of *Artemisia annua* extracts against species of the *Fusarium* genus, primarily in the context of agricultural control of *Fusarium* and other root-associated pathogenic consortia [23,24]. In another study, authors evaluated *A. annua* against *Fusarium* spp. at concentrations as low as 4 μL/mL. The chemical composition of the extract may confer advantages due to the presence of camphor, borneol, deoxyartemisinin, and β-caryophyllene oxide [25]. Nevertheless, it is important to note that previous studies have emphasized that ART, a constituent of *A. annua* leaves, represents only a minor fraction of the total herbal biomass—sometimes as low as 0.01% of the dry weight [26]. This low abundance is a critical factor when considering treatment strategies. Therefore, although several recent efforts have focused on treating *F. solani* with *A. annua* extracts, we demonstrate here that ART alone is sufficient to produce a significant antifungal effect.

In the present work, the antifungal effect of ART was evaluated and shown to significantly reduce the growth of *Fs* within 24 h compared with negative controls. To our knowledge, this is the first report not only demonstrating the effect of ART on *Fs*. but also examining its intracellular localization within the fungal nucleus. Artemisinin’s antifungal activity is greatly ROS-mediated: homolytic cleavage of its endoperoxide linkage generates reactive ROS that attack cellular lipid bilayers and proteins. ROS-mediated oxidative stress of membrane structure and protein activity leads to killing of fungal cells. In keeping with such paradigm, confocal imaging of propidium iodide (PI)–labeled *Fs* conidia treated with ART demonstrated large-scale membrane permeabilization, while protein carbonylation assays reflected an extensive increase in oxidatively modified protein. Together, these results prove that broad-scale membrane destruction as well as protein oxidation by ART-radicals are responsible for the potent cytotoxic antifungal activity observed. These data are in keeping with those obtained by confocal and carbonylation studies. Taken together, these findings suggest that ART warrants further evaluation in animal models, such as murine systems, to assess its potential as a therapeutic candidate, given its favorable chemical properties and prospects for future clinical trials in humans.

## 4. Materials and Methods

### 4.1. Fungal Strain

The *Fs* isolate employed in this study was originally obtained from infected plant tissue, as described previously [8]. The human-pathogenetic fungal strain were rest isolated from 13 non-immunocompromised or-non immunosuppressed patients with keratomycosis and authorization were obtained by courtesy of the hospital at “El Hospital para Prevenir la Ceguera en México, Luis Sánchez Bulnes” among January/2013 to August/2016. Patients from nine Mexican states. The isolate was purified, taxonomically verified through morphological assessment and internal transcribed spacer rDNA sequencing, and routinely propagated on Potato Dextrose Agar (PDA) at 25 ± 2 °C. Subcultures were maintained twice per week to ensure viability and phenotypic stability.

### 4.2. Compound Preparation and Fungal Inhibition Assay

ART, a sesquiterpene lactone originally isolated from Artemisia annua, was purchased commercially from Sigma-Aldrich (St. Louis, MO, USA) and used without further purification. The compound was freshly prepared prior to each experiment and handled under light-protected conditions to preserve stability. As previously reported by our group, a 750 μM stock solution of artemisinin (ART; >95% purity, HPLC-verified) was prepared in phosphate buffer (pH 7.4), from which working dilutions of 500, 350, 200, 150, and 75 μM were generated. In parallel, distilled water and 0.5% dimethyl sulfoxide (DMSO) were prepared as negative growth controls. All solutions were sterilized by vacuum filtration using a polyethersulfone (PES) membrane filter (0.2 μm pore size; Sartorius, Göttingen, Germany). A 10 mL suspension of *Fs* conidia, standardized to 2 × 10^6^ colony-forming units (CFU)/mL, was used to evaluate the fungicidal and/or fungistatic effects of ART and controls.

Two experimental conditions were established: (1) immediate plating (0 h exposure), and (2) 24 h exposure at 25 ± 2 °C prior to plating. For the non-exposure condition, 1 mL of each ART dilution or control was mixed with the *Fs* conidial suspension and plated directly onto PDA in Petri dishes, followed by incubation at 25 ± 2 °C for 24 h. For the exposure condition, ART–*Fs* suspensions were incubated at 25 ± 2 °C for 24 h prior to plating under identical conditions.

All experiments were conducted in triplicate and independently repeated three times. Following incubation, CFUs were quantified for each treatment and condition using a digital colony counter in combination with a MiniLight Box (Upland, CA, USA).

### 4.3. Confocal Scanning Laser Microscopy 

To assess fungal membrane integrity after ART treatment, propidium iodide (PI; 1 mg/mL) was added to cell suspensions exposed to ART for 24 h at 25 ± 2 °C. PI uptake was used as a marker of membrane compromise. Samples were analyzed using a confocal scanning laser microscope (Leica DMI 6000) equipped (Leica, Wetzlar, Germany) with a 543 nm helium–neon laser for fluorescence excitation (Leica TCS-SP8) (Leica, Wetzlar, Germany).

### 4.4. Carbonylation Assay

*Fs* cultures were initiated at a density of 1 × 10^6^ cells in 15 mL medium and incubated to the logarithmic phase. Cell density was determined by direct counting with a hemocytometer. Cells were washed twice with sterile phosphate-buffered saline and centrifuged at 2000× *g*. The pellet was resuspended in LIT complete medium to a final density of 4 × 10^5^ cells/mL. A 500 μL aliquot of each suspension was transferred to a 96-well plate for incubation and treatment.

Cells were incubated for 24 h at 28 °C under 150 μM artemisinin dissolved (<1% DMSO) and fluconazole (FLZ, 32 μg/mL ≈ 104.5 μM, water stock adjusted to <1% DMSO in-well). Vehicle for negative control was treated with only <1% DMSO. H_2_O_2_ (100 μM) was used for positive control of oxidation. Two negative controls ultrapure Milli-Q water and Milli-Q water with dodecyl bisulfate (each equivalent to <1% DMSO) were employed. The percentage of DMSO was made equivalent for all the conditions. Triplicate wells per condition were treated for 24 h at 28 °C. All conditions were assayed thrice in triplicate and repeated three times independently.

Following incubation, cells were lysed and carbonyl groups quantified using 2,4-dinitrophenylhydrazine (DNPH) derivatization. Equal volumes (1 mL each) of cell lysate and 20 mM DNPH in 1.5 M HCl were mixed and incubated at 25 °C. Protein precipitation was achieved with 20% trichloroacetic acid, followed by chilling on ice for 15 min and centrifugation at 12,500× *g* for 10 min. The protein pellet was washed twice with ethanol:ethyl acetate (1:1 *v*/*v*), dissolved in 2 mL of 8.5 M guanidine-HCl (pH 7.4), and vortexed. Absorbance was measured at 370 nm, and carbonyl content was calculated using a molar extinction coefficient of 22,000 M^−1^ cm^−1^, in accordance with Hartley’s method.

## 5. Conclusions

In summary, ART significantly inhibited the growth of FS within 24 h, with evidence of intracellular localization and oxidative damage potentially driven by endoperoxide cleavage. Carbonyl accumulation confirmed ROS generation as the core fungicidal mechanism. These findings identify ART as a promising antifungal candidate for in vivo validation and potential clinical development.

## Data Availability

The original contributions presented in this study are included in the article. Further inquiries can be directed to the corresponding author.

## Abbreviations

The following abbreviations are used in this manuscript:

ART	Artemisinin
*Fs*	*Fusarium solani*
